# Supplementary material for: A set of multi-entry identification keys to African frugivorous flies (Diptera, Tephritidae)
Source: Zookeys. 2014 Jul 24;(428):97–108. doi: 10.3897/zookeys.428.7366 (PMC4143993; doi:10.3897/zookeys.428.7366)
Supplement: Supplementary material 6 — Key to Ceratitis [file zookeys-428-097-s006.zip › SF6_ZooKeys_key to Ceratitis/key/SF6_ZooKeys_key to Ceratitis/Media/Html/Ceratitis grahami .htm]

Ceratitis (Ceratalaspis) grahami Munro


***Ceratitis*** **(*Ceratalaspis*)
*grahami* Munro**

*Ceratitis* (*Pardalaspis*) *grahami*
Munro, 1935: 303.

Body
length: 5.00 mm; wing length: 5.40 mm.

Male

Head.
Antenna dark orange. Antennal inplant with darker horizontal band (but not very
clear). Third antennal segment twice as long as second segment. Arista with
hairs of moderate length over entire length (but not plumose). Frons with short
scattered hairs which are largely the same colour as frons; convex, in lateral
view not distinctly projecting forwards at antennal implant. Chaetotaxy normal
for subgenus except only one frontal bristle.

Thorax.
Postpronotum white, with small black spot. Ground colour of mesonotum pale with
orange tinge. Chaetotaxy normal for subgenus. Scapular setae pale. One
anepisternal bristle. Scutellum white basally, otherwise yellow with three
black separate markings apically; basally without dark spots, only slight
darker colouration. Subscutellum completely dark.

Legs
yellow; setation typical for subgenus, mainly pale especially on femora. Fore
femur with dense orange setation ventrally and posterodorsally along entire
length, not outspoken feathery but more bushy; similar setation on dorsal and
ventral side along apical fourth of hind femur.

Wing
bands with markings extensively yellow. Banding, marginal band continuous;
cubital band free and medial band absent; crossvein r-m at middle of discal
cell. Crossvein dm-cu posteriorly more outwards than anteriorly.

Abdomen.
Brownish colour. Setation and banding typical for subgenus.

Female

Unknown.

(Description
after De Meyer, 1998)
